# Movable Kidney—II

**Published:** 1908-03-14

**Authors:** 


### MOVABLE KIDNEY.—II.

It was said in last week's article that a movable kidney is constantly associated with a greater or lesser degree of neurasthenia; and it is this which makes the disease a difficult one both to diagnose and to treat. The first symptom complained of is abdominal pain, often of a vague nature and wide distribution. In cases in which the patient has been informed that the kidney is movable this may be referred to the renal region. But if his attention has not been focussed to that organ, he may describe the pain as being situated in one or more parts of the abdomen quite remote from this. There can, of course, be little doubt that there are genuine cases in which pain is definitely due to the mobility of the kidney. This is most easily explained either on the ground that the displacement of the kidney causes torsion of its vessels or of the corresponding ureter, and that this leads to engorgement, or that the branches of the sympathetic plexus are dragged upon. The pain is often associated with vomiting; and here again two possible explanations may account for it; either, as in the former case, that it is due to traction on the sympathetic plexus, or that there is some mechanical interference with the stomach itself. We may reasonably suppose that the latter is, at any rate, a possible occurrence, since jaundice is an occasional complication, and this is undoubtedly due to torsion of, or pressure on, the common bile duct.

From time to time the patient experiences periodical exacerbations of all the symptoms, and the pain in a genuine case may become so severe as to simulate an attack of renal colic. These paroxysms are associated with a diminished excretion of urine owing to kinking of the ureter. The name of Dietl's crisis has been given to this phenomenon. In some cases there is also a definite tenderness on palpation over the kidney; and in a woman this may be referred to the uterus or ovary.

The clinical course varies largely. The patient may go on for years in much the same condition, getting no better and no worse; or, if there is genuine mobility of the kidney, a definite hydronephrosis may result from the obstruction to the flow of urine, owing to the kinking of the ureter. This, however, is not common. In other cases the nervous symptoms get the upper hand, and the patient becomes a chronic invalid or, more rarely, develops a degree of mental instability sufficient to render him certifiable. The following is a case in point:—A woman, aged forty-six, complained that for four years she had had attacks of terrible pain in the epigastrium, accompanied by vomiting immediately after food. The average duration of the attacks was ten days, and she generally had about one month's freedom from pain between the attacks. She had never been jaundiced. She had had five children, the youngest of whom was fourteen. All the labours had been normal. She was a typical neurasthenic, and took delight in reeling off a long recital of all her symptoms, and became somewhat irritable if she was interrupted. She had a definitely palpable tumour in the right side of the abdomen, and there was very little doubt that this was a displaced kidney. She was a most unreasonable patient, and disliked anything being done for her by the nurses. She continued in this state for some days, and then was apparently seized with acute mania. She left her bed and made straight for the window. Arrived there, she broke two panes of glass, and was only restrained from hurting herself with extreme difficulty. From this time forward she was quite uncontrollable, screaming and shouting at the top of her voice, and it was eventually found necessary to remove her to an asylum.

Treatment resolves itself into palliative and radical. It may be said at once that no form of radical treatment should be considered if there are marked symptoms of neurasthenia. It is worse than useless, for, apart from the admitted uncertainty of the results of nephrorrhaphy, the operation merely serves still further to concentrate the patient's attention on his kidney, and his last state is likely to be worse than his first. Nor should any operation be performed if it is considered that the dropping of the kidney is part of a generalised enteroptosis; for it is obvious that surgical measures cannot benefit the patient. In either of these cases a well-fitting abdominal belt should be applied, and its efficacy may be increased by fixing on its internal aspect a triangular air-cushion to press the displaced kidney backwards and upwards. Its mechanical action is probably of very little value, but in a neurasthenic it may do an astonishing amount of good by mere suggestion—that is to say that if he can only be persuaded that the belt is keeping his kidney in position his symptoms may almost entirely disappear.

In the absence of advanced neurasthenia or of enteroptosis, and if palliative treatment has failed, it is justifiable to attempt to fix the kidney in its place by surgical measures. Several methods of doing this have been described, but the following gives the most satisfactory results. The kidney is exposed by the ordinary nephrectomy incision and drawn into the wound. A cruciform incision is made into the capsule on its posterior aspect, so that four angular flaps of capsule can be raised. These are sutured with a sufficient number of fine silk sutures to the edges of the cut lumbar aponeurosis and muscles. Plenty of capsule must be taken up in the stitches. The wound is closed in the ordinary way, and no drainage tube need be inserted. It is quite unusual for there to be any troublesome hæmorrhage. This method is much preferable to the others, which have been described. Some surgeons, for instance, sling the kidney to the posterior abdominal wall by stitches passed through its substance, while others advocate merely exposing it and plugging the wound, so that the organ becomes fixed by granulation tissue: but the latter method exposes the patient to the risk of a lumbar hernia; and against the former it may be said that it is always wise to avoid putting stitches into the kidney if possible, because they tend to cut through as soon as they are tied. The results of the operation are not uniformly good, but they are sufficiently encouraging to render the proceeding quite justifiable in properly selected cases.

